# Effect of Vitamin D Deficiency on Arterial Stiffness in Pregnant Women with Preeclampsia and Pregnancy-Induced Hypertension and Implications for Fetal Development

**DOI:** 10.3390/biomedicines12071595

**Published:** 2024-07-18

**Authors:** Mircea Iurciuc, Florina Buleu, Stela Iurciuc, Izabella Petre, Daian Popa, Radu Dumitru Moleriu, Anca Bordianu, Oana Suciu, Rabia Tasdemir, Ramona-Elena Dragomir, Madalina Otilia Timircan, Ion Petre

**Affiliations:** 1 Department of Cardiology, “Victor Babes” University of Medicine and Pharmacy, E. Murgu Square No. 2, 300041 Timisoara, Romania; iurciuc.mircea@umft.ro (M.I.); iurciuc.stela@umft.ro (S.I.); 2 County Emergency Clinical Hospital “Pius Brinzeu”, 300732 Timisoara, Romania; petre.izabella@umft.ro (I.P.); rabia.tasdemir@gmail.com (R.T.); madalina-otilia.timircan@umft.ro (M.O.T.); 3 Department XII of Obstetrics and Gynaecology, “Victor Babes” University of Medicine and Pharmacy, 300041 Timisoara, Romania; 4 Doctoral School, “Victor Babes” University of Medicine and Pharmacy, 300041 Timisoara, Romania; daian-ionel.popa@umft.ro (D.P.); petre.ion@umft.ro (I.P.); 5 Department of Surgery, Emergency Discipline, “Victor Babes” University of Medicine and Pharmacy, 300041 Timisoara, Romania; 6 Department of Functional Sciences, Medical Informatics and Biostatistics Discipline, “Victor Babes” University of Medicine and Pharmacy, 300041 Timisoara, Romania; radu.moleriu@umft.ro; 7 Department of Plastic Surgery and Reconstructive Microsurgery Bagdasar-Arseni, Emergency Hospital Bucharest, University of Medicine and Pharmacy “Carol Davila”, 010825 Bucharest, Romania; anca.bordianu@gmail.com; 8 Department of Microbiology, “Victor Babeș” University of Medicine and Pharmacy, Eftimie Murgu Sq. No. 2, 300041 Timisoara, Romania; suciu.oana@umft.ro; 9 Doctoral School, “Carol Davila” University of Medicine and Pharmacy, 020021 Bucharest, Romania; ramona.dragomir@drd.umfcd.ro

**Keywords:** vitamin D deficit, arterial stiffness, preeclampsia, pregnancy-induced hypertension, developmental disorders in the fetus

## Abstract

Background and objectives: Over the past few years, researchers have focused on the importance of vitamin D in the health of pregnant women and in reducing the chances of developmental disorders occurring in fetuses. In addition, a link has been established between fetal development and arterial stiffness in hypertensive disorders that occur during pregnancy. Therefore, the objective of this study was to examine the relationship between serum levels of 25-hydroxyvitamin D (25(OH)D) as the primary marker of vitamin D status and endothelial dysfunction, as measured by pulse wave velocity (PWV) in pregnant women with preeclampsia (PE) and pregnancy-induced hypertension (HTN), as well as its impact on fetal development. Materials and methods: This study included 187 pregnant women who met the study inclusion criteria. Pregnant women were divided into two groups: pregnancy-induced hypertension (HTN group), which included 100 patients (53.48%), and preeclampsia (PE group), which included 87 patients (46.52%). Results: Significant differences regarding the augmentation index (Aix) brachial, PWVao, heart rate, and systolic or diastolic blood pressure with more increased values were observed for the HTN group vs. the preeclampsia group in the current research (*p* < 0.001). Additionally, the Aix brachial index was significantly lower in the preeclampsia group compared to the HTN group (1.76 ± 0.71 for the HTN group vs. 0.62 ± 0.5 for the preeclampsia group, *p* < 0.001). A severe matern serum 25(OH)D level deficiency was associated with a more severe subcategory of prematurity (*p* < 0.001) and with increased chances of newborn preterm birth (*p* < 0.05). Moreover, the negative effect of severe maternal serum 25(OH)D level deficiency was studied for each group regarding the blood pressure values, Aix brachial, PWVao values in the second and third trimesters, and fetus weight. The Kruskal–Wallis test was applied for this, obtaining significant differences in all cases: open paren p less than 0.05 and closed. When serum severe 25(OH)D levels deficiency was present, arterial stiffness parameters were significantly worse. Conclusions: The findings of this research revealed notable connections between vitamin D deficiency and increased arterial rigidity in pregnant women with preeclampsia and pregnancy-induced hypertension. These results emphasize the significance of conducting both examinations to obtain a more comprehensive evaluation of these patients. Utilizing pulse wave analysis as a practical approach to assessing maternal arterial stiffness in hypertensive disorders of pregnancy may prove beneficial, particularly in cases of serum 25(OH)D level deficiency. It could play a key role in identifying patients at higher risk of worsening disease severity and, thus, preventing any impact on fetal development.

## 1. Introduction 

One prevalent public health problem, vitamin D deficiency, often goes unnoticed and remains untreated [1]. This deficiency has been associated with an increased risk of various health disorders, including hypertension, diabetes, metabolic syndrome, cancers, congestive heart failure, autoimmune diseases, and chronic vascular inflammation [1,2,3]. 

A unique category, however, is pregnant women, who experience subtle changes in their metabolism and endocrine functions. The nutritional intake, which must meet the needs of both the pregnant woman and the developing fetus [4], also impacts circulating 25(OH)D concentrations, with lower plasma concentrations in vegetarians and vegans compared to meat and fish consumers [5]. Unfortunately, because of rapid changes in modern lifestyles, the prevalence of lower serum 25(OH)D concentrations during pregnancy has increased and become a global concern [6]; therefore, evidence that supports the effective use of vitamin D supplementation is important [7]. Extensive clinical research has proved the crucial role of vitamin D in the well-being of pregnant women and newborns. Deficient levels of this vitamin during pregnancy can have detrimental effects on the health of both the mother and the pregnancy itself, including hypertensive disorders of pregnancy, premature rupture of membranes, premature delivery [6], and gestational diabetes mellitus [8,9].

Furthermore, vitamin D deficiency during pregnancy can significantly impact the growth and development of the fetus, particularly in terms of bone development, potentially leading to adverse effects on weight [10,11]. The Rostami et al. study also found that preterm birth was not only associated with vitamin D deficiency but was also indirectly related to low blood levels of 25(OH)D and increased risks [12]. Therefore, circulating 25(OH)D serum levels are considered the primary marker of prenatal vitamin D status. 

Vitamin D deficiency has been linked to arterial stiffness, a well-known indicator of cardiovascular disease and a marker of subclinical atherosclerosis [1]. In pregnant women, arterial stiffness is a crucial measure of vascular health that has shown promising results in predicting the occurrence of hypertensive disorders during pregnancy, such as preeclampsia and pregnancy-induced hypertension [13]. A recent comprehensive analysis and meta-analyses in this field have revealed an elevation in arterial stiffness indices among women who develop hypertensive disorders during pregnancy, emphasizing the potential of measuring arterial stiffness as an early predictive marker [14]. Evidence is accumulating that arterial stiffness measurements can identify women who will later develop hypertensive disorders during pregnancy [15]. 

The significance of vitamin D in developing endothelial dysfunction is widely recognized. However, its role as a contributing factor to arterial stiffness in pregnancy-induced hypertension and preeclampsia, particularly in Romania, where information on vitamin D deficiency during pregnancy is limited, has not been thoroughly examined. This study aimed to assess if vitamin D deficiency is related to arterial stiffness (measured by pulse wave velocity) in pregnant women with preeclampsia and pregnancy-induced hypertension and if there is a correlation between lower serum 25(OH)D levels and fetal development. 

## 2. Material and Methods 

### 2.1. Study Design and the Selection of Participants 

The study was carried out in the Obstetrics and Gynaecology Departments of the Emergency Clinical Hospital “Pius Brînzeu” in Timișoara and included 187 pregnant women who met the study inclusion criteria. Before participating, all patients gave informed consent per the principles outlined in the Declaration of Helsinki. The Emergency Clinical Hospital Ethics Committee “Pius Brînzeu” Timișoara approved this study (decision no. 54/20 April 2017). 

Our study focused exclusively on pregnant women between 18 and 45 who were in their first or second pregnancy and had stable clinical and hemodynamic conditions. Only those in the second trimester of pregnancy and non-smokers were eligible for inclusion. To ensure the integrity of our study’s design, we excluded women with a history of arterial hypertension, intrauterine fetal growth restriction, placental abruption, or those taking medication that could potentially impact blood pressure. Additionally, we excluded individuals with multiple pregnancies, coronary artery disease, valvular heart disease, congenital heart disease, heart failure, cardiomyopathy, arrhythmias or conduction disorders on electrocardiography, chronic kidney or liver diseases, cancer, a history of alcohol or drug abuse, hematological disease, ongoing infection, systemic inflammatory conditions, or any autoimmune disease such as systemic lupus erythematosus or antiphospholipid antibody syndrome. Furthermore, we excluded individuals with hyperlipidemia, pre-existing diabetes mellitus, hypercholesterolemia, peripheral arterial disease, thyroid function abnormalities, significant anemia (defined as a hemoglobin level of 9 g/dL or less), or known psychiatric comorbidities. Lastly, pregnancies with aneuploidy or significant fetal abnormalities were also excluded from our study.

Pregnant women were divided into two groups: pregnancy-induced hypertension (HTN group), which included 100 patients (53.48%), and preeclampsia (PE group), which included 87 patients (46.52%). The patients from both groups were selected with similar characteristics: age, body mass index (BMI), environment, and habits.

No pregnant women in the study had taken vitamin D supplements independently of calcium and other multivitamin/micronutrient supplements. Therefore, that season, to not influence the serum 25(OH)D level, only pregnant women from whom the blood sampling was taken between March 1 and November 1 were included in the study. Moreover, anamnestically, none of the pregnant women were exposed to excessive sunlight due to the cardiologist’s recommendations. 

### 2.2. Newborns Assessment

The classification of preterm birth, according to the World Health Organization (WHO), is based on the duration of gestation. Specifically, any birth that occurs before completing 37 weeks of gestation or fewer than 259 days since the woman’s last menstrual period (LMP) is considered preterm. They are subdivided based on gestational age (GA) to categorize preterm births further. The widely accepted definition of preterm birth categorizes it into three groups: extremely preterm (less than 28 weeks), very preterm (28 to less than 32 weeks), and moderate to late preterm (32 to 37 weeks). This classification is recognized and commonly used in the field [16]. 

The Apgar score is a standardized assessment of a newborn’s condition immediately after birth. It consists of five parts. Each category is weighted equally and assigned a 0, 1, or 2 value. The components are then added to produce a score recorded 1 and 5 min after birth. A score from 7 to 10 is considered safe, 4 to 6 is moderately abnormal, and 0 to 3 is considered low in term and late preterm infants [17]. 

### 2.3. Preeclampsia and Pregnancy-Induced Hypertension Definitions 

The criteria for diagnosing preeclampsia were established based on the guidelines provided by the International Society for the Study of Hypertension in Pregnancy (ISSHP) [18]. To meet this definition, women who were previously normotensive must have a systolic blood pressure of 140 mmHg or higher and a diastolic blood pressure of 90 mmHg or higher on two separate occasions, at least four hours apart after reaching the 20-week mark of gestation. Additionally, proteinuria of 300 mg or more in a 24-h urine collection or two readings of at least ++ on dipstick analysis of midstream or catheter urine specimens (if a 24-h collection is unavailable) is required alongside hypertension.

Pregnancy-induced hypertension, also known as gestational hypertension, is a condition characterized by high blood pressure, specifically a systolic BP of 140 or higher and a diastolic BP of 90 or higher, that develops after 20 weeks of pregnancy. This condition occurs without proteinuria or abnormalities in blood tests or hematological markers. It is important to note that gestational hypertension typically does not coincide with restrictions in fetal growth [18]

### 2.4. 25-Hydroxyvitamin D Analysis 

The DIAsource 25-OH Vitamin D Total Elisa 90kit by DIAsource Immunoassays SA in Louvain-la-Neuve, Belgium, was explicitly used to determine the serum levels of 25(OH)D. Reproducibility and accuracy were evaluated with two low and high concentrations samples. Their concentrations were measured in 10 different runs in duplicate for reproducibility and a single run for repeatability precision. According to the company’s application notes, we used the Free 25OH Vitamin D serum control to calibrate and validate the measurements. The limit of blank (LOB) was calculated to be 2.012 ng/mL. The limit of detection (LOD) was calculated as the LOB—1.645 standard deviation of a low-concentration sample tested in 10 different runs. The LOD was calculated to be 3.035 ng/mL. The interpretation of the values is as follows: levels equal to or greater than 30 ng/mL are considered sufficient, levels between 21 ng/mL and 29 ng/mL are deemed insufficient, mild deficiency is defined by levels between 10 ng/mL and 20 ng/mL, moderate deficiency is indicated by levels greater than five ng/mL but less than ten ng/mL, and severe deficiency is defined by levels less than five ng/mL [19]. 

### 2.5. Measurement of Arterial Stiffness 

According to the European Society of Cardiology, arterial stiffness can be accurately assessed using pulse wave velocity, the gold standard [20]. This study conducted carotid-femoral PWV measurements using the MedexpertArteriograph device version 3.0.0.3, manufactured by TensioMedKft., Budapest, Hungary. This device determined the central pressure and augmentation index and measured pulse wave propagation velocity in the aorta (PWVao). To ensure the utmost precision, pregnant participants were instructed to lie supine for 10 min in a quiet room before the measurements. The readings obtained from this accredited medical device included central pressure pulse (PP), augmentation index, and pulse wave propagation velocity in the aorta. Following the guidelines outlined in the expert consensus document on arterial stiffness, a single examiner conducted multiple measurements using the prescribed determination method. To ensure accurate results, participants were required to abstain from smoking, caffeine-containing foods, or drinks for at least 3 h before the measurements. Additionally, alcohol consumption was prohibited for at least 10 h before the investigation. Similar to blood pressure, arterial diameter and stiffness exhibit a circadian rhythm, with an increase observed during sleep. As a result, participants were instructed to stay awake during the assessment to avoid interfering with these parameters. Throughout the measurements, participants maintained silence. According to multiple sources, the accepted measurement for pulse wave velocity in adult females is typically 7.4 m/s. However, it is essential to acknowledge that younger, physically active women generally have lower baseline values, averaging around 6.1 m/s (with a range from 4.6 to 7.5 m/s). As women grow older, these values gradually rise due to various factors such as changes in the vascular endothelium, elevated blood pressure, related health conditions, and even pregnancy [21,22]. 

### 2.6. Statistical Analysis 

The data were gathered using the Microsoft Excel 2021 program; the JASPv18.3 program was used for the statistical analysis. After the inclusion and exclusion criteria were applied, 187 patients underwent the study, split into two groups: patients with hypertension or preeclampsia. The Shapiro–Wilk test was used to obtain a non-normal distribution for testing the data distribution; for this, only non-parametrical tests were used in the following analysis. The Mann–Whitney test was applied to determine whether the two groups’ differences were significant. When more than two different groups are analyzed, the Kruskal–Wallis test is used. The Pearson coefficient (r) and the Spearman coefficient (R) were calculated to test the association between variables, and a linear correlation/regression model was applied to determine the significance of the data. The significance was set at α=0.05 for the whole study. 

## 3. Results

The mean values of the numerical variables were tested by applying a Mann–Whitney test, obtaining significant differences in the quantity of serum 25(OH)D levels for mother and newborn, Aix brachial: [%], PWVao [m/s], in the second and third trimester, heart rate (beat/minute) (HR), systolic (mmHg) (Sys) and diastolic (mmHg)(Dia) blood pressure, gestational period (weeks), APGAR score, fetus weight (grams); and insignificant differences (*p* > 0.05) or mother age and mother body mass index. The entire analysis is presented in Table 1. The analyzed parameters have significantly above-average values in the HTN group. 

**Figure 1:**
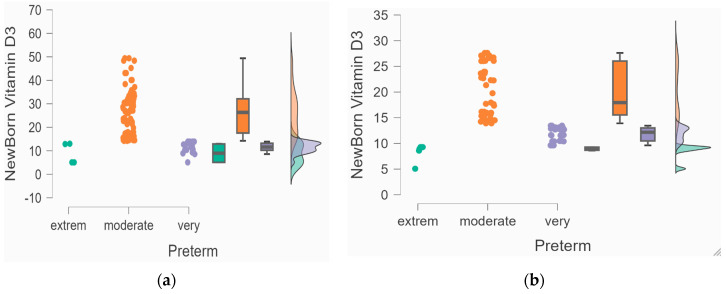
The data from the Kruskal–Wallis test regarding the relation between serum 25(OH)D level and the prematurity subcategory in both groups are represented using raincloud plots: (**a**) The relation between the serum 25(OH)D level and the prematurity subcategory in the HTN group. (**b**) The relation between the serum 25(OH)D level and the prematurity subcategory in the preeclampsia group.

The newborn serum 25(OH)D levels in each group were compared based on the prematurity stage using a Kruskal–Wallis test. We observed severe deficient serum levels associated with a more severe prematurity subcategory. Further, vitamin D deficiency in pregnant women was associated with premature birth. Moreover, when the linear correlation model was applied, after calculating the Spearman coefficient, a significant, strong, positive, and direct association was obtained (for the HTN group, a *p* < 0.001, r = 0.78 and *p* < 0.001, r = 0.83 for the preeclampsia group). In addition, newborns with severe lower serum 25(OH)D levels were highly premature, with even lower levels when they were birthed by pregnant women with preeclampsia (Figure 1). 

A linear regression model was applied in both groups to test the possible association between the lower serum 25(OH)D levels in mothers and their newborns, between mothers and their blood pressure values, between lower serum 25(OH)D levels in newborns and their weight on birth, and between lower serum 25(OH)D levels in mothers and their age or BMI. 

A significant, robust, and positive association was found in the case of serum 25(OH)D level dependence between mother and newborn ($\left|r\right|>0.75,\;p<0.05$); a weak positive association was obtained between serum 25(OH)D level and the mother’s systolic blood pressure ($\left|r\right|<0.25,\;p<0.05$); a week true median negative indirect significant correlation was obtained between serum 25(OH)D level in newborns and their weight at birth. All the results are presented in Table 2.

Moreover, for each group, the negative effect of lower serum 25(OH)D level on the mother’s body was studied regarding the blood pressure values, Aix brachial [%], PWVao [m/s] values in the second and third trimesters, and fetus weight. The Kruskal–Wallis test was applied for this, obtaining significant differences in all cases (*p* < 0.05). When a severe serum 25(OH)D level was present, the analyzed parameters worsened significantly.

## 4. Discussion

The occurrence of hypertensive disorders during pregnancy, such as preeclampsia and pregnancy-induced hypertension, continues to be a significant complication with an impact on both mother and fetus all over the world [18]. Ensuring optimal healthcare for the prevention and treatment of these disorders in pregnant women remains a top priority in overall health policy [23,24], as they are known to be one of the leading causes of maternal deaths globally [25,26,27,28,29]. Early prediction of these disorders is crucial to effectively monitor at-risk pregnant women and prevent the development of serious complications. Current clinical guidelines, both nationally and internationally, recommend assessing a combination of maternal risk factors, placental biomarkers, and arterial stiffness to predict the risk of preeclampsia and pregnancy-induced hypertension [20,30,31]. However, the accuracy of these markers is limited due to high variability in detection rates and false positives, especially during early pregnancy [32]. There is a growing interest in utilizing arterial stiffness measurements as a predictive tool during pregnancy, as they have shown the ability to detect changes in vessel hemodynamics that differentiate normotensive and non-normotensive pregnancies [14,21]. 

Significant differences in hemodynamic and arterial stiffness parameters were observed between pregnant women groups (HTN group vs. preeclampsia group) in the current research (*p* < 0.001). These parameters include Aix brachial, PWVao, heart rate, and systolic or diastolic blood pressure. Pregnant women from the HTN group exhibited higher values for these parameters than the preeclampsia group. Additionally, the Aix brachial index was significantly lower in the preeclampsia group compared to the HTN group (1.77 ± 0.71 for the HTN group vs. 0.62 ± 0.5 for the preeclampsia group, *p* < 0.001). This index reflects the aorta’s ability to receive blood. In a healthy pregnancy, there is an increase in compliance in the arterial system. However, our study found that women with pregnancy-induced hypertension did not demonstrate this physiological adaptation when compared to pregnant women with preeclampsia.

Previous studies have also shown peripheral and aortic arterial stiffness in individuals with preeclampsia [33,34]. It has been observed that pregnant women who experienced hypertensive disorders during pregnancy exhibit increased arterial stiffness postpartum [35]. However, when Kim et al. conducted their study, they discovered no variation in the longitudinal alteration of arterial stiffness between women with preeclampsia and those with normal blood pressure within a year after delivery [36]. To the best of our knowledge, this is the initial evidence indicating that pregnancy-induced hypertension leads to greater arterial stiffness compared to preeclampsia, as demonstrated by our results. No other research from the literature compared these two hypertensive disorders. One study assesses arterial stiffness in pregnancies complicated by hypertensive disorders: preeclampsia and chronic hypertension. Women with chronic hypertension exhibited elevated systemic vascular resistance, similar to those with preeclampsia. However, the impact on arterial stiffness was comparatively lesser in cases with chronic hypertension [37].

Evidence suggests that increased arterial stiffness plays a role in the development of early gestational age in normotensive pregnant women. This indicates a connection between fetal growth and maternal endothelial function [38]. When comparing pregnancies affected by hypertensive disorders with and without a small gestational age neonate, it was found that both groups had significantly higher Aix measured by PWV compared to uncomplicated normotensive pregnancies. The highest Aix values were observed in pregnancies complicated by hypertensive disorders and a small for gestational age neonate, indicating a worsening of arterial stiffness with a more severe clinical presentation [39]. These findings likely indicate reduced vascular compliance and increased systemic vascular resistance in pregnancies affected by preeclampsia or pregnancy-induced hypertension.

The severity of hypertensive disorders of pregnancy can be indicated not only by the gestational age at which preeclampsia or pregnancy-induced hypertension occurs but also by the presence of fetal growth restriction [40]. Our findings reveal a significant correlation (*p* < 0.05) between serum 25(OH)D level deficiency in the mother and newborn, accompanied by a low APGAR score and fetal growth restriction. These results provide compelling evidence for the link between serum 25(OH)D level deficit and the severity of preeclampsia and pregnancy-induced hypertension. 

A noteworthy finding from our study is a statistically significant correlation between serum 25(OH)D level deficiency in newborns and their birth weight, indicating a negative indirect relationship (*p* < 0.001). This observation aligns with previous research in the field. For instance, Mahfod et al. [41] reported a significant decrease in fetal weight among pregnant women with fetal growth restriction, consistent with the findings of Khalessi et al. [42], who suggested that maternal vitamin D deficiency could elevate the risk of low birth weight in neonates.

This study found that maternal serum vitamin D levels in highly preterm infants decreased significantly. This coincides with research by Zhao et al., who demonstrated an inverse relationship between maternal 25-hydroxyvitamin D concentrations and the likelihood of low birth weight, preterm birth, and early gestational age. However, their meta-analysis found no significant association between maternal vitamin D levels and macrosomia or intrauterine growth restriction [43].

Since the results have been inconsistent, there should be prudence when interpreting observational studies examining the relationship between vitamin D and preeclampsia. Many of these studies considered factors such as maternal age, body mass index, season, and gestational trimester at the time of sample collection. Some studies, including our own, also exclude smoking pregnant women as a variable [44], and others bring evidence suggesting that pregnant women who smoke have lower levels of 25(OH)D regardless of sun exposure season [45]. Benachi et al. conducted a study that found no significant association between vitamin D insufficiency in the first trimester and preeclampsia. However, they discovered that women with sufficient vitamin D levels during the third trimester and those who maintained sufficient levels in both the first and third trimesters had a significantly reduced risk of developing preeclampsia [46]. 

The results of our study emphasized the potential of measuring arterial stiffness as an early predictive tool in pregnancy together with serum 25(OH)D level. It is becoming increasingly evident that these measurements can effectively identify women who will develop hypertensive disorders later on.

We also believe that further research is crucial to understanding the impact of vitamin levels throughout pregnancy. New research should focus on detecting deficiencies in the early stages of pregnancy, specifically in the first weeks, and determining if these deficiencies can be swiftly restored to normal levels. By doing so, we can potentially improve pregnancy outcomes and address arterial stiffness, which is linked to restricted fetal growth in patients with hypertensive disorders during pregnancy. 

## 5. Study Limitations

However, it is essential to note that this type of research had a few limitations. It requires a large sample size and an adequate number of participants. Additionally, it would be beneficial to exclusively study populations with a multi-generational history of preeclampsia or pregnancy-induced hypertension in each pregnancy. This targeted approach would provide valuable insights into how vitamin D influences arterial stiffness in this specific group of pregnant women. It is worth mentioning that previous studies, including the reviewed literature, have primarily focused on apparently healthy groups without documented comorbidities [8]. However, it is crucial to acknowledge that these comorbidities have been identified as significant disease burdens over the years [47]. Furthermore, due to the fact that the period of data collection in this study did not comprise all seasons (only from 1 March to 1 November), we could not account for seasonal variability. However, it appears that the influence of seasonality on UV radiation is more pronounced in regions with high latitudes and limited exposure to sunlight in winter, as opposed to lower latitudes [48], which is the region where this study was carried out. Figueiredo et al. performed adjusted multiple analyses to determine if seasons have an impact on serum 25(OH)D concentrations: they observed that pregnant women who started the study in spring (*p* < 0.001), winter (*p* < 0.001), or fall (*p* = 0.028) showed a longitudinal increase, whereas women who started the study in summer did not [49]. 

## 6. Conclusions

We demonstrated that in pregnant women with preeclampsia and pregnancy-induced hypertension, increased arterial stiffness may be involved, in part, in the pathogenesis of preterm birth, suggesting an association between fetal development and maternal endothelial dysfunction. Pulse wave analysis may be a clinically applicable method for assessing maternal arterial stiffness. It may be more relevant to intrauterine fetal growth when associated with lower serum 25(OH)D levels. 

## Figures and Tables

**Table 1:** Descriptive statistics were run between the studied groups. The *p*-value was obtained after applying a Mann—Whitney test, obtaining significant differences in open paren *p* less than 0.05 and close paren in most cases. The essential results are highlighted in grey.

Variables	Group	N	Mean	SD	SE	Coefficient of Variation	*p* Value
**Mother’s age, years**	HTN	100	37.56	2.65	0.26	0.07	*p* = 0.989
	Preeclampsia	87	37.58	2.65	0.28	0.07	
**Mother serum 25(OH)D level, ng/mL**	HTN	100	18.77	7.29	0.73	0.39	*p* < 0.001
	Preeclampsia	87	13.85	6.46	0.69	0.47	
**Newborn serum 25(OH)D level, ng/mL**	HTN	100	22.77	10.89	1.09	0.47	*p* < 0.001
	Preeclampsia	87	16.48	5.96	0.64	0.36	
**BMI (kg/m^2^)**	HTN	100	30.33	2.71	0.27	0.09	*p* = 0.861
	Preeclampsia	87	30.41	2.70	0.29	0.09	
**Aix brachial: [%] Trim II**	HTN	100	1.76	0.71	0.07	0.41	*p* < 0.001
	Preeclampsia	87	0.62	0.50	0.05	0.80	
**Aix brachial: [%] Trim III**	HTN	100	4.09	1.37	0.14	0.34	*p* < 0.001
	Preeclampsia	87	3.08	0.94	0.10	0.31	
**PWVao [m/s] Trim II**	HTN	100	11.79	1.01	0.10	0.07	*p* < 0.001
	Preeclampsia	87	9.85	0.86	0.09	0.09	
**PWVao [m/s] Trim III**	HTN	100	13.43	1.15	0.12	0.09	*p* < 0.001
	Preeclampsia	87	11.07	1.23	0.13	0.11	
**HR [beats/min]**	HTN	100	95.79	8.31	0.83	0.09	*p* < 0.001
	Preeclampsia	87	84.08	6.17	0.66	0.07	
**Sys [mmHg]**	HTN	100	208.40	16.49	1.65	0.08	*p* < 0.001
	Preeclampsia	87	180.92	11.29	1.21	0.06	
**Dia [mmHg]**	HTN	100	129.69	9.18	0.92	0.07	*p* < 0.001
	Preeclampsia	87	110.70	5.85	0.63	0.05	
**Gestational period [weeks]**	HTN	100	34.24	3.19	0.32	0.09	*p* < 0.001
	Preeclampsia	87	32.72	2.99	0.32	0.09	
**APGAR score [at 1 min]**	HTN	100	7.61	2.14	0.21	0.28	*p* = 0.007
	Preeclampsia	87	7.03	1.94	0.21	0.28	
**Fetus weight [gram]**	HTN	100	2953.70	528.86	52.87	0.18	*p* < 0.001
	Preeclampsia	87	1989.89	714.63	76.62	0.36	

**Table 2:** The regression analysis uses the numerical variables from the study. The Pearson coefficient was calculated using the sample to determine the direction of the association. The significant results are highlighted in grey.

Association between	Results	
	HTN Group	Preeclampsia Group
serum 25(OH)D level in mothers and newborn	r=0.75, p<0.001	r=0.91, *p* < 0.001
serum 25(OH)D level in mothers and systolic blood pressure	r=0.23, p=0.047	r=0.27, *p* = 0.013
serum 25(OH)D level in mothers and diastolic blood pressure	r=0.12, p>0.05	r=0.17, p>0.05
serum 25(OH)D level in newborns and their weight at birth	r=−0.26, p=0.02	r=−0.38, *p* < 0.001
serum 25(OH)D level in mothers and their age	r=−0.09, p>0.05	r=−0.11, p>0.05
serum 25(OH)D level in mothers and their BMI	r=−0.16, p>0.05	r=−0.13, p>0.05

## Data Availability

The datasets are not publicly available, but de-identified data may be provided upon request by the corresponding author.
